# Choroidal measurements in decision making for retinopathy of prematurity: a decision tree analysis

**DOI:** 10.1186/s40942-023-00520-6

**Published:** 2024-01-11

**Authors:** Mohammadreza Mehrabi Bahar, Farhad Salari, Afsar Dastjanifarahani, Fariba Ghassemi, C. Armitage Harper, Fatemeh Bazvand

**Affiliations:** 1 Retina Services, Eye Research Center, Farabi Eye Hospital, Tehran University of Medical Sciences, Qazvin Square, South Kargar Street, Tehran, Iran; 2 Austin Retina Associates, University of Texas-San Antonio, Austin, USA

**Keywords:** Retinopathy of prematurity, Spectral domain optical coherence tomography, Choroidal thicknesses, Choroidal vascular index, Intravitreal bevacizumab

## Abstract

**Background:**

To compare the choroidal thickness and vascular profile of premature infants with ROP (retinopathy of prematurity) using a handheld SD-OCT device.

**Methods:**

We performed horizontal SD-OCT scans through the fovea in 115 eyes of 66 premature infants. Premature infants included 2 groups [infants with ROP requiring treatment (as treatment group) vs. infants without ROP or with ROP not- requiring treatment (as no-treatment group)] Choroidal thicknesses (CT) were measured at 5 points, including the fovea, 250 µm, and 500 µm mm nasal and temporal to the fovea. The choroidal vascularity index (CVI) and choroidal stromal index (CSI) were also calculated. The classification and regression tree (CRT) algorithm was used to predict the need for treatment based on all OCT characteristics.

**Results:**

Mean CT was higher in 500 µm nasal to the fovea compared to temporal CT (275.8 ± 64.8 and 257.1 ± 57.07, P value < 0.03). No statistically significant difference was found regarding CVI, corrected CVI, and temporal and nasal CT in the treatment group versus the no-treatment group. The foveal CT was significantly lower in ROP patients with the plus disease compared to not-plus ROP (P value = 0.03. ANOVA, Bonferroni posthoc test). CT was not significantly different between plus and pre-plus patients (P-value = 0.9, ANOVA, Bonferroni posthoc test). No significant relationship was found between the stage of ROP and choroidal thickness (P value > 0.05, GEE). The decision tree analysis showed that in infants with ROP, the most important predictor for the need for treatment is CSI.

**Conclusion:**

This study delineated the possible effectiveness of choroidal measurements as an additive to decision-making for ROP. We also demonstrated that choroidal involution is associated with the presence of plus disease, not with the stage of ROP. We demonstrated that choroidal measurements are very sensitive but not specific tools for assessing the need for treatment in ROP patients.

## Background

Retinopathy of prematurity (ROP) is a major cause of treatable visual loss in the pediatric population [1, 2]. The main pathogenesis behind this disease is the exposure of the immature retina to relative hyperoxia, resulting in the reduction of growth factors [3–6]. Although retinal neovascularization was the primary suspect for the visual loss, the revelation of central photoreceptor loss raises a probable role of choroidal vasculature [7–9]. Due to limited human specimens for pathologic examination as a result of the increased survival rate of premature infants, oxygen-induced retinopathy (OIR) models in the rats were utilized by scientists to enhance our knowledge about ROP pathogenesis [10]. Using OIR models, it has been shown that choroidal involution can be found in ROP patients [7]. As the choroid is responsible for supplying the oxygen demands of the highly metabolically active outer retinal layers [11], the choroidal vasculature involution might be responsible for photoreceptor loss in ROP patients [7].

With the advent of handheld Ocular coherence tomography, a growing body of literature evaluates choroidal parameters in ROP [12–14]. Choroidal thinning was detectable in patients with a history of treatment for ROP and also spontaneously regressed ROP [15, 16]. Therefore, choroidal involution may be responsible for permanent vision loss in these patients. Despite many studies evaluating OCT characteristics in ROP, none of them considered its usage in planning for the treatment of these patients. In this study, we used a decision tree analysis to find whether choroidal parameters could be used as a marker to determine the need for treatment.

## Method

### Subjects

This cross-sectional case–control study was performed in Farabi Eye Hospital, Tehran University of Medical Science between 2019 and 2021. This study adhered to the tenets of the Declaration of Helsinki and was approved by the local ethics committee of the Tehran University of Medical Sciences (https://ethics.research.ac.ir/IR.TUMS.FARABIH.REC.1400.065). Informed consent was obtained from all the parents or the legal guardians of the involved infants. We evaluated 156 eyes of 81 premature neonates. All infants were born between 2019 and 2021.

**Fig. 1:**
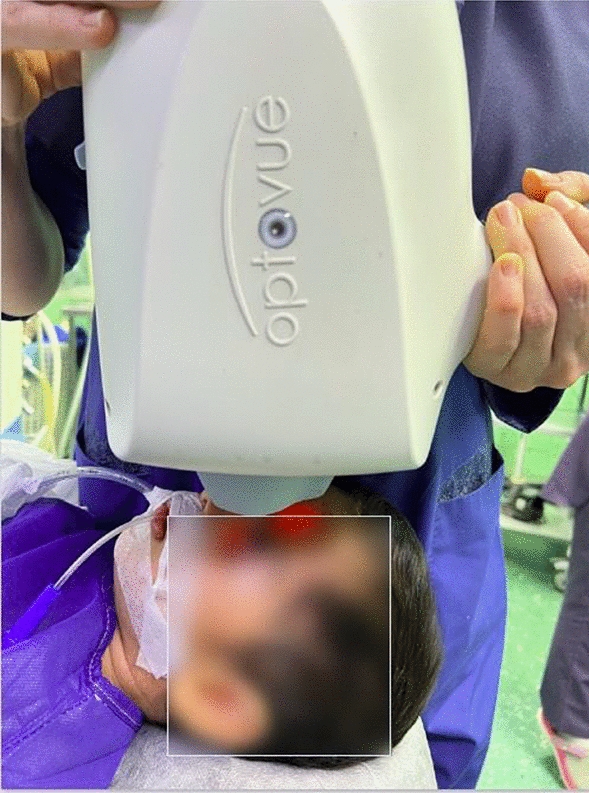
A handheld portable optical coherence tomography (OCT) image of an 18-month-old infant with Familial Exudative Vitreoretinopathy (FEVR) who underwent imaging under general anesthesia

The ROP diagnosis and staging were performed using indirect ophthalmoscopy by two ROP experts (FB and AD) [17]. The preterm infants with type 1 ROP or more severe received treatment [18]. One patient (one eye) who was a candidate for the laser treatment (zone III with confluent stage 3) received Intravitreal bevacizumab as his general health condition did not allow him to get general anesthesia. The patients who received general anesthesia or sedation for treatment were included in this study as a treatment group. They were examined using handheld portable SD-OCT (Optovue iVue SD-OCT Wellness report; Optovue Corporation, Fremont, CA) after pupil dilatation and before treatment (Fig. 1). Some preterm infants were examined in the neonatal intensive care unit (NICU), and routinely they received sedation for fundus examination with sclera depression for controlling stress and pain in addition to topical anesthesia. The patients in the NICU who did not require treatment were chosen as a control group (no-treatment group), and OCT was performed for them.

We have three sedation protocols: 1. In IVB treated group, the sedation protocol was oral sucrose 0.5–1 mg/kg 2 min before IVB injection and repeated 2 min later. 2. In NICU-admitted infants (control group or no-treatment group), the sedation protocol was nasal administration of ketamine 2–3 mg/kg plus fentanyl 2–3 µg/kg. 3. In the laser-treated group, IV ketamine 1 mg/kg with suppository acetaminophen 20 mg/kg was used, and if needed, ketamine 1 mg/kg was added. In cases that were not sedated with this protocol, sevoflurane 3% with oxygen through a mask for 2–3 min was administered.

Intravitreal injections were performed under topical anesthesia and light sedation due to the general condition. The OCT was performed for them. After administration of 10% povidone-iodine for periocular skin and 5% povidone-iodine for ocular surface, half of the adult doses of bevacizumab (0.625 mg/0.025 mL) were injected with a 30-gauge needle 1–1.5 mm behind the limbus into the vitreous cavity. Topical gentamycin or sulfacetamide was given for three days post-injection. In cases that need laser ablation, the indirect laser was performed in avascular areas using a confluent or near-confluent pattern with moderate intensity after general anesthesia and full pupillary dilation. Topical gentamycin or sulfacetamide, topical mydrax 0.5%, and topical betamethasone were prescribed for seven days in patients receiving indirect laser.

In order to control imaging artifacts, the examination without any sedation or general anesthesia and images with low quality were excluded. Patients with unstable general conditions or hazy media, such as corneal edema, were also excluded. ROP staging, zone, and presence of plus were recorded. Of 156 eyes, 133 required treatment (treatment group). Twenty-two eyes required Bevacizumab injection, and the other 111 eyes received indirect laser photocoagulation.

### Image processing

**Fig. 2:**
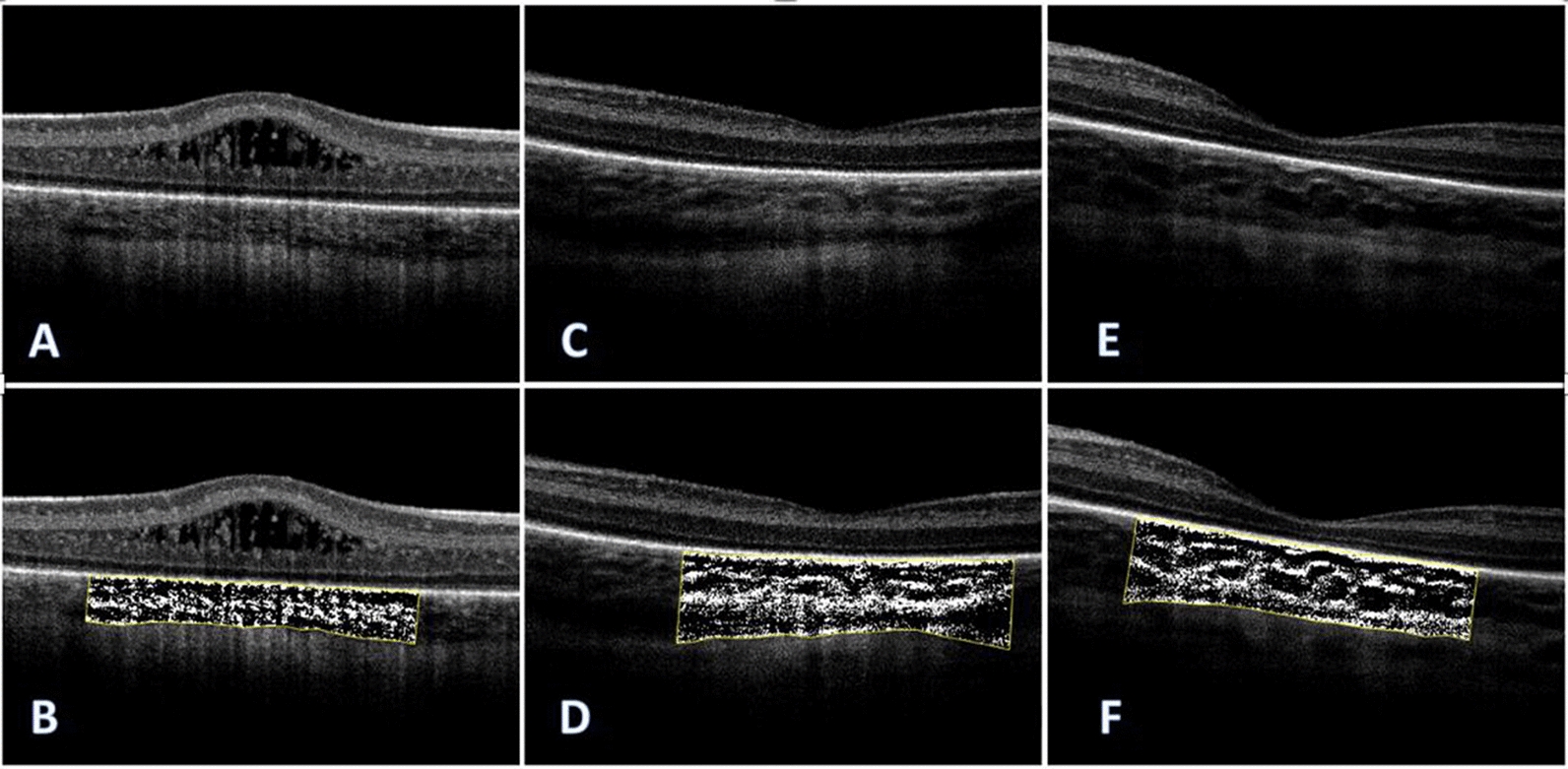
**A** Optical coherence tomography (OCT) image of ROP infant who received IVB and **B** the same patient’s Choroidal Vascularity Index (CVI) image. **C** OCT image of ROP laser-treated patient and **D** CVI image of the same patient. **E** ROP patient who received no treatment and **F** CVI image of the same one

OCT scans were obtained by a single experienced examiner. Details of the imaging technique and OCT quality evaluations are available in our previous publication [17] 0.115 eyes from 66 cases had OCT imaging with sufficient quality for choroidal measurements. Choroidal thickness (CT) was defined as the distance from Bruch’s membrane to the inner part choroidoscleral junction. Choroidal thickness was calculated at the subfoveal, 250 µm, and 500 µm nasal and temporal to the fovea manually using ImageJ software (National Institutes of Health, Bethesda, MD, USA) (Fig. 2). The average of these calculations was considered foveal choroidal thickness (FCT). As previously described, Agrawal et al. protocol was applied to imaging to calculate luminal area (LA), stromal area (SA), and choroidal vascularity index (CVI) [19]. We also calculated the choroidal stromal index (CSI) by subtracting CVI from 1 (CSI = 1 − CVI).

All of the choroidal parameters were measured by an experienced co-author (MRMB). To evaluate the observer variability, the measurement was re-analyzed two times for 100 cases, and the average values were used for the analysis. To evaluate the intra-observer reliability of the CVI measurements, we calculated the intraclass correlation coefficient (ICC). The ICC was 0.95 with a 95% confidence interval (CI) of 0.91 to 0.97 (p < 0.001) (excellent reliability).

### Statistical analysis

We described choroidal parameters using mean (standard deviation) and median (interquartile range). We used the generalized estimating equation (GEE) to assess the correlation of the ROP severity and the choroidal thickness with consideration of the possible correlation of measurements in bilateral samples. In the comparison of treatment type and no-treatment group, multiple comparisons were considered by the Sidak method. We used a decision tree analysis to obtain a method for the prediction of the needs for treatment. The classification and regression tree (CRT) approach was used to grow the tree. The maximum depth of the tree was set at five levels, and the minimum number of cases of the parent node and the child node was set to 20 and 5, respectively. In addition, the sensitivity, specificity, and diagnostic accuracy of the model were reported. The inter-grader reliability for each parameter was also calculated using the intraclass correlation coefficient (ICC). All statistical analysis performed by SPSS (IBM Corp. Released 2017. IBM SPSS Statistics for Windows, Version 25.0. Armonk, NY: IBM Corp).

## Results

### Patient demographics

Of the 156 eyes who underwent OCT evaluation, 115 eyes (66 cases) had imaging with sufficient quality for choroidal measurement. Of 95 eyes that underwent treatment, 12 received intravitreal injections of Bevacizumab, and 83 were treated with laser photocoagulation. The findings were compared to 20 eyes of ROP children who didn’t require treatment. The demographic features of the study population are presented in Table 1**.**

**Table 1:** Baseline characteristics of patients included in the study

Variable	Label	Need to treat			Treatment type		
		No	Yes	Sig.	IVB	Laser	Sig.
Sex	Female	14 (70%)	45 (47%)	0.06	7 (58%)	38 (46%)	0.41
	Male	6 (30%)	50 (53%)		5 (42%)	45 (54%)	
Postmenstrual age	Mean ± SD	42.3 ± 7.8	38.7 ± 3.24	0.15	35.6 ± 5.29	39.25 ± 2.52	0.001
Gestational age	Mean ± SD	31.2 ± 4.5	29.35 ± 1.83	0.29	28 ± 1.28	29.5 ± 1.82	0.008
Birth weight	Mean ± SD	1508 ± 590	1285 ± 337	0.1	1121.67 ± 181	1310 ± 349	0.08
Treatment with O_2_	Yes	1 (5%)	83 (87%)	0.001	10 (83%)	73 (88%)	0.65
Mechanical ventilation	Yes	1 (5%)	35 (37%)	0.005	8 (67%)	27 (33%)	0.02
Sepsis	Yes	0 (0%)	7 (7%)	0.21	1 (8%)	6 (7%)	0.89
Jaundice	Yes	1 (5%)	38 (40%)	0.003	5 (42%)	33 (40%)	0.90
Blood transfusion	Yes	0 (0%)	36 (38%)	0.001	5 (42%)	31 (37%)	0.77
Anemia	Yes	0 (0%)	18 (19%)	0.03	3 (25%)	15 (18%)	0.56
Gestational diabetes mellitus	Yes	0 (0%)	18 (19%)	0.03	2 (17%)	16 (19%)	0.82
Zone	I	0 (0%)	9 (9%)	0.001	4 (33%)	5 (6%)	0.001
	II	10 (50%)	81 (85%)		3 (25%)	78 (94%)	
	II posterior	0 (0%)	4 (4%)		4 (33%)	0 (0%)	
	III	5 (25%)	1 (1%)		1 (8%)	0 (0%)	
	Complete vascularization	5 (25%)	0 (0%)		0 (0%)	0 (0%)	
Aggressive posterior ROP		0 (0%)	0 (0%)		0 (0%)	0 (0%)	
Stage	0	5 (25%)	0 (0%)	0.001	0 (0%)	0 (0%)	0.02
	1	9 (60%)	1 (1%)		1 (10%)	0 (0%)	
	2	0 (0%)	5 (6%)		0 (0%)	5 (6%)	
	3	0 (0%)	76 (86%)		9 (90%)	67 (86%)	
	4	0 (0%)	6 (7%)		0 (0%)	6 (8%)	
Plus stage	No plus	17 (100%)	1 (1%)	0.001	1 (10%)	0 (0%)	0.02
	Pre-plus	0 (0%)	15 (17%)		0 (0%)	15 (19%)	
	Plus	0 (0%)	72 (82%)		9 (90%)	63 (81%)	

*IVB* intravitreal bevacizumab

The Choroidal thickness measurements in the horizontal foveal scan are displayed in Tables 2 and 3. In all the subjects, the highest choroidal thickness was in the subfoveal region (temporal: P value < 0.05 and nasal: P value < 0.05. t-test). The mean CT was higher in 500 µm nasal to the fovea in comparison with temporal CT (275.8 ± 64.8 and 257.1 ± 57.07, P value < 0.03, t-test). However, at the 250-µm eccentricity, there wasn’t any significant difference between nasal and temporal borders.

**Table 2:** Comparison of choroidal parameter in ROP-treated and not-treated ROP patients

Variable	Need to treat		Diff.	95% CI (confidence interval)		P value
	No	Yes		Lower	Upper	
Total area						
Mean ± SD	349 ± 73	438 ± 114	− 86.4	− 145.4	− 27.3	0.005
Median (range)	350 (250 to 480)	440 (210 to 810)				
Stromal area						
Mean ± SD	282 ± 61	313 ± 72	− 31.0	− 69.4	7.4	0.112
Median (range)	284 (179 to 381)	309 (182 to 528)				
Luminal area						
Mean ± SD	67 ± 23	124 ± 76	− 55.7	− 93.8	− 17.6	0.005
Median (range)	71 (32 to 102)	101 (26 to 340)				
Choroidal vascular index (CVI)						
Mean ± SD	81 ± 6	73 ± 11	7.4	1.7	13.1	0.012
Median (range)	80 (72 to 91)	75 (48 to 92)				
Choroidal stromal index (CSI)						
Mean ± SD	19 ± 6	27 ± 11	− 7.4	− 13.1	− 1.7	0.012
Median (range)	20 (9 to 28)	25 (8 to 52)				
Foveal choroidal thickness (FCT)						
Mean ± SD	254 ± 56	305 ± 83	− 43	− 85.8	− 0.7	0.04
Median (range)	245 (170 to 390)	300 (130 to 480)				
Temporal choroidal thickness 500 (TCT 500)						
Mean ± SD	238 ± 55	263 ± 57	− 24.6	− 52.5	3.4	0.084
Median (range)	(147 to 323)	(128 to 433)				
Temporal choroidal thickness 250 (TCT250)						
Mean ± SD	243 ± 49	272 ± 61	− 28.5	− 57.5	0.5	0.054
Median (range)	246 (152 to 309)	274 (135 to 461)				
Sub-foveal choroidal thickness (SFCT)						
Mean ± SD	247 ± 53	284 ± 66	− 36.1	− 67.7	− 4.5	0.026
Median (range)	250 (152 to 330)	290 (132 to 462)				
Nasal choroidal thickness 250 (NCT 250)						
Mean ± SD	239 ± 49	284 ± 68	− 44.5	− 76.8	− 12.3	0.007
Median (range)	236 (135 to 314)	289 (108 to 435)				
Nasal choroidal thickness 500 (NCT500)						
Mean ± SD	249 ± 54	282 ± 66	− 31.7	− 63.2	− 0.1	0.049
Median (range)	249 (138 to 334)	283 (152 to 487)				

*SD* standard deviation

**Table 3:** Comparison of choroidal parameters in IVB-treated and laser-treated ROP patients

Variable	Treatment		Diff.	95% CI (confidence interval)		P value
	IVB	Laser		Lower	Upper	
Area						
Mean ± SD	398 ± 84	444 ± 117	− 54.6	− 128.0	18.7	0.142
Median (range)	370 (310 to 520)	440 (210 to 810)				
Stromal area						
Mean ± SD	265 ± 61	321 ± 71	− 52.7	− 98.1	− 7.3	0.024
Median (range)	269 (187 to 394)	316 (182 to 528)				
Luminal area						
Mean ± SD	133 ± 54	123 ± 79	− 2.8	− 52.5	46.9	0.910
Median (range)	126 (45 to 202)	97 (26 to 340)				
Choroidal vascular index (CVI)						
Mean ± SD	67 ± 12	74 ± 11	− 4.1	− 11.4	3.1	0.259
Median (range)	65 (53 to 86)	75 (48 to 92)				
Choroidal stromal index (CSI)						
Mean ± SD	32 ± 12	26 ± 11	4.1	− 3.1	11.4	0.259
Median (range)	35 (14 to 47)	25 (8 to 52)				
Foveal choroidal thickness (FCT)						
Mean ± SD	274 ± 63	309 ± 86	− 38.8	− 85.5	7.8	0.1
Median (range)	248 (200 to 420)	300 (130 to 580)				
Temporal choroidal thickness 500 (TCT 500)						
Mean ± SD	234 ± 43	268 ± 58	− 31.2	− 64.9	2.6	0.070
Median (range)	(194 to 322)	(128 to 433)				
Temporal choroidal thickness 250 (TCT 250)						
Mean ± SD	245 ± 51	276 ± 62	− 30.2	− 66.2	5.9	0.100
Median (range)	227 (200 to 370)	280 (135 to 461)				
Sub-foveal choroidal thickness (SFCT)						
Mean ± SD	260 ± 57	288 ± 67	− 27.5	− 67.1	12.1	0.171
Median (range)	242 (200 to 410)	298 (132 to 462)				
Nasal choroidal thickness 250 (NCT250)						
Mean ± SD	258 ± 72	289 ± 68	− 34.4	− 75.1	6.3	0.096
Median (range)	230 (197 to 433)	295 (108 to 435)				
Nasal choroidal thickness 500 (NCT500)						
Mean ± SD	258 ± 91	286 ± 62	− 31.8	− 71.1	7.5	0.111
Median (range)	230 (168 to 487)	290 (152 to 423)				

*SD* standard deviation, *IVB* intravitreal bevacizumab

Significant differences were found between the treatment and control group only regarding the total area and luminal area after Sidak correction for multiple comparisons (total area: 438 ± 114 and 349 ± 73, luminal area: 124 ± 76 and 67 ± 23 in treatment and control groups retrospectively all P value < 0.0051) (Table 2). No statistically significant difference was found regarding CVI, corrected CVI, and temporal and nasal CT in children with ROP receiving treatment versus the control group. In the treatment group, there was not any significant difference between the laser and IVB groups regarding choroidal parameters after Sidak correction for multiple comparisons.

The foveal CT was significantly lower in ROP patients with the plus disease compared to non-plus ROP (P value = 0.03. ANOVA, Bonferroni posthoc test). CT was not significantly different between plus and pre-plus patients (P-value = 0.9, ANOVA, Bonferroni posthoc test). No significant relationship was found between the stage of ROP and choroidal thickness (P value > 0.05, GEE).

### Decision tree analysis

**Fig. 3:**
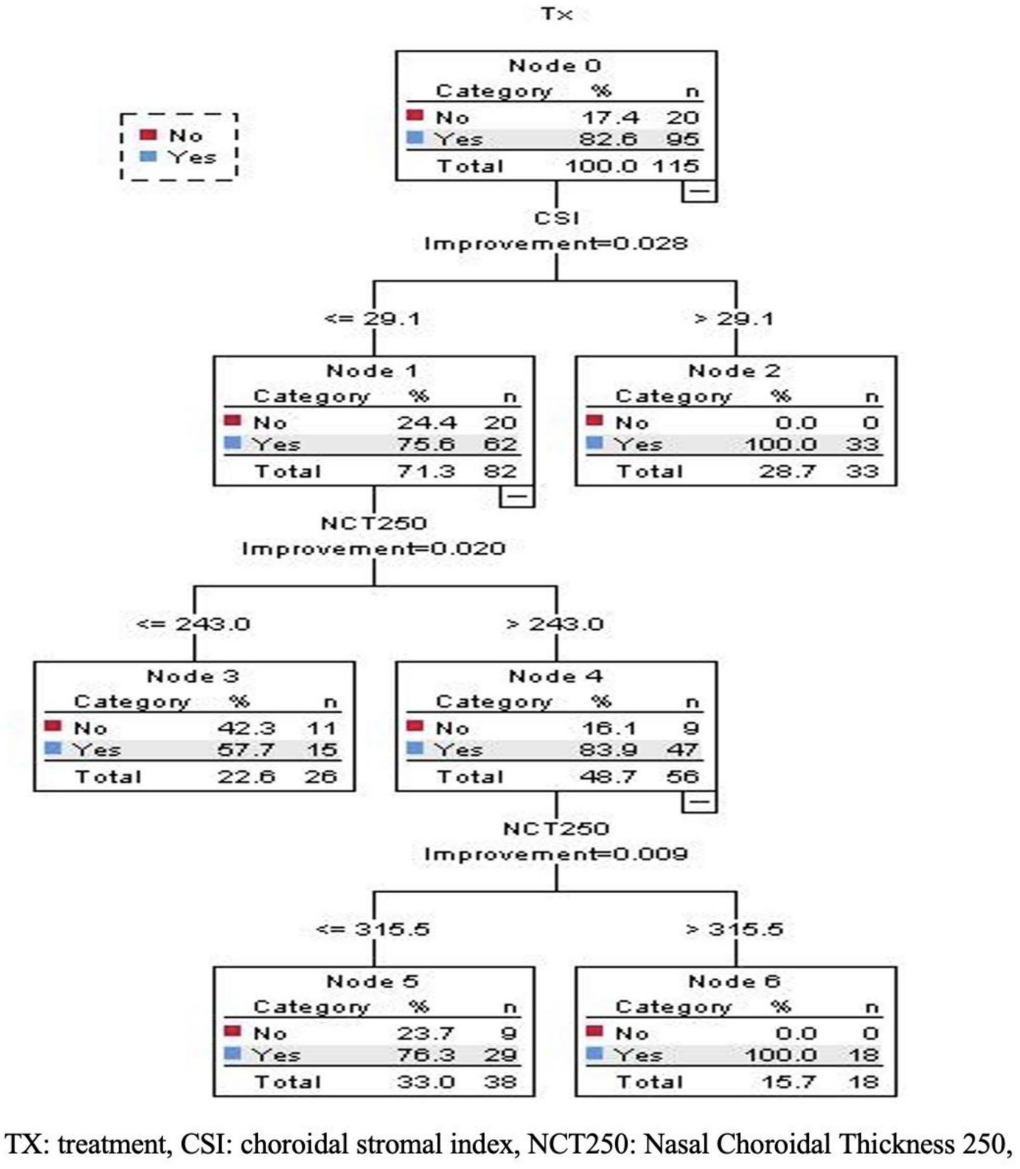
Decision tree based on choroidal parameter

CRT algorithm was used to predict the need for treatment based on all OCT characteristics. The tree diagram (Fig. 3) shows tree construction based on the test sample of 115 cases. There are seven nodes that consist of 4 terminal nodes, and the depth of the tree is 3. The Parent node has 20 absences (17.4%) and 95 presence (82.6%) of indication for treatment. The first discriminator, “CSI, ” splits the root node into two child nodes: low avascularity (less than or equal to 29.1) (node 1, n = 82) and high (more than 29.1) (terminal node 2, n = 33). The improvement for this classification is 0.028. The next discriminator is the mean choroidal thickness in 250 µm of nasal to the fovea “NCT250”, split into low choroidal thickness (less than or equal to 243, terminal node 3, n = 26) and high choroidal thickness (more than 243, node 4, n = 56). The improvement for this classification is 0.020. When the nasal choroidal thickness is more than 243, then the final discriminator is again NCT 250 (0.009 improvement), which produces two terminal nodes, 5 and 6. Percentages in each category and each joint category are shown in Fig. 3. The predictors: SFCT, CVI, CT 500, and CT 250 didn’t contribute to the classification tree used. The prediction accuracy over the entire sample size was 82.6%, with a sensitivity of 100% and a specificity of 0%. The misclassification rate was 0.174, with a standard error of 0.035.

## Discussion

The current study aimed to assess the choroidal profile in treatment–requiring ROP neonates and compare it with ROP neonates not requiring treatment. Our study showed that foveal choroidal thickness was significantly lower in ROP patients with the plus disease compared to non-plus ROP (P value = 0.03. ANOVA, Bonferroni posthoc test). However, CT was not significantly different between plus and pre-plus patients (P-value = 0.9, ANOVA, Bonferroni posthoc test). We found no significant relationship between the different staging of ROP and choroidal thickness. It might be explained partly by that choroidal vasculature especially choriocapillaris might be involved other than choroidal thickness. The assessment of choriocapillaris is very difficult in premature infants.

Retinopathy of prematurity (ROP) is considered a disease of retinal vascularization; however, recent evidence demonstrates that choroidal vasculature abnormality is also important and is responsible for outer retinal dysfunction and visual loss [20]. Several clinical studies have demonstrated choroidal thinning associated with ROP in older children and adults using OCT imaging [13–16]. Anderson and colleagues reported lower subfoveal choroidal thickness in children and young adults with a history of ROP treatment with laser ablation and/or cryotherapy compared with healthy controls [21]. Similar results have been shown by Bowl et al. in a study that compared choroidal thickness in 17 young children with a history of treated ROP or spontaneously regressed ROP. They also showed that reduced choroidal thickness was linked to ROP severity [16]. However, few studies in the literature evaluated choroidal thickness in infants with ROP [12, 13, 22]. Erol and his colleagues studied subfoveal choroidal thickness in 80 premature infants. They found that the thickness of the choroid decreased with the severity of ROP [13]. In another study by Mangalesh et al., it is shown that the presence of pre-plus/plus disease versus without accompanied by thinner choroid [23]. In consensus with previous studies [23], we also demonstrated the choroid was thinner in ROP neonates with the plus disease compared to not-plus disease. It has been suggested that the thinning of the choroid in ROP neonates may be due to oxidative stress and choroidal vascular loss [7, 12, 24, 25]. This choroidal vascular loss has been proven angiographically. In a study by Islam et al., the presence of choroidal hypo fluorescence in the central and or peripheral retina of ROP patients was demonstrated [26].

Interestingly, our results indicated that the choroidal thickness didn’t significantly differ in ROP neonates requiring treatment (tROP) in comparison with not treatment requiring group (nROP). A previous study by Erol and colleagues showed that choroidal thickness in ROP patients with a grade 2 or 3 was lower than grade 0. However, they showed no significant difference between the choroidal thickness of grade 1 and grade 2/3 ROP patients [13]. These findings agree with our results, as 60% of the untreated group was stage 1 ROP, and 92.7% of the treated group was grade 2/3 ROP. Also, Mangalesh et al. reported thinner choroid was associated with pre-plus or plus disease and lower gestational age and birth weight but not the ROP stage [23].

Consistent with previous studies, we found that choroidal involution is associated with the presence of pre-plus/plus, not with the ROP stage.

According to our data, the choroidal vascularity index (CVI) and choroidal stromal index (CSI) weren’t significantly different in the treatment and untreated groups. CVI is a novel means of choroidal evaluation introduced by Agrawal et al. in 2016 [19]. It has been shown that this parameter is less variable than the choroidal thickness and less influenced by systemic and ocular circumstances [19]. This marker is rarely studied in ROP patients. Consistent with our results, Lavric et al. reported CVI preterm children aged 5–15 years had the same CVI compared to preterm children with a history of ROP [27].

For the first time, we used the decision tree method to analyze the use of choroidal parameters to treat ROP patients. In contrast to more traditional statistics, such as linear regression, the decision tree method analyzed the nonlinear and interactive patterns between factors. This study successfully used decision tree analysis to detect the most important choroidal factor for identifying the treatment group and determining cut points for each parameter. The results presented in Fig. 3 showed that in ROP patients, the most important choroidal factor for predicting the need for treatment was CSI. All subjects, associated with CSI higher than 29.1, required treatment. This novel index represents the avascularity of the choroid. Therefore, patients with a higher value might benefit from treatment to stop the vicious circle of oxidative stress in ROP patients. Further studies are required to evaluate the addition of choroidal parameters to the current practice of ROP screening regarding effectiveness and outcomes.

The current study focused on the choroidal parameters; this does not exclude the importance of retinal parameters in ROP. This study has other limitations. The handheld OCT imaging was time-consuming and prone to imaging artifacts; future handheld OCT with a faster imaging acquisition would improve their imaging quality and feasibility. Also, the application of newer technologies like enhanced depth OCT or OCT angiography may gather more information about the choroid. We encourage further research using these newer devices to enhance our knowledge about the role of the choroid in ROP management. We measured choroidal measurements manually, and therefore, although reliability analysis showed acceptable observer agreement, the measurements might be susceptible to imprecision. We have a small number of ROPs that did not require treatment. Also, the role of gestational age and preexisting systemic and neurological disorders in choroidal parameters were not evaluated, and future studies should be planned to evaluate these factors.

## Conclusion

This study delineated the possible effectiveness of choroidal measurements as an additive to decision-making for ROP. We also demonstrated that choroidal involution is associated with the presence of plus disease, not with the stage of ROP.

## Data Availability

Our datasets analyzed during the current study are available from the corresponding author on request.

## Abbreviations

ROP: Retinopathy of prematurity
SD-OCT: Spectral domain optical coherence tomography
IVB: Intravitreal bevacizumab
CT: Choroidal thicknesses
CVI: Choroidal vascular index
CSI: Choroidal stromal index
